# Penile Lymphangioma: review of the literature with a case presentation

**DOI:** 10.1186/s12610-018-0081-3

**Published:** 2019-01-28

**Authors:** Mohamed Macki, Sharath Kumar Anand, Hayan Jaratli, Ali A. Dabaja

**Affiliations:** 1 Department of Neurosurgery, Henry Ford Hospital, 2799 West Grand Blvd, Detroit, MI 48202 USA; 2 Department of Pathology, Henry Ford Hospital, 2799 West Grand Blvd, Detroit, MI 48202 USA; 3 Department of Vattikuti Urology Institute, Henry Ford Hospital, 2799 West Grand Blvd, Detroit, MI 48202 USA

**Keywords:** Cavernous, Circumscriptum, Cystic, Hygroma, Lymphangioma, Lymphangiectasis, Lymphangiectasia, Penile, Penis

## Abstract

**Background:**

Penile lymphangiomas are rare manifestations of lymphangiomas or lymphatic malformations which are more commonly found in the head or neck region of the body. Lymphangiomas are further categorized as lymphangioma circumscriptum, cavernous lymphangioma, cystic hygroma, or acquired lymphangiomas (also known as lymphangiectasia), based on their depth and etiology.

**Results:**

A literature review revealed only 30 cases of penile lymphangioma between 1947 and March 30, 2018. Several causes were attributed to the acquired penile lymphangiomas, including trauma, phimosis, and infection. While penile lymphangiomas can be initially mistaken for an infection, a thorough history and physical examination is sufficient to clinically diagnose a lymphangioma of the penis. Historically, surgical excision has been the gold standard of treatment for this condition. When asymptomatic, patients may opt for conservative management with avoidance of mechanical trauma alone. Other physicians have revealed novel treatment plans to rid patients of their penile lymphangioma such as a staged laser procedure.

**Conclusion:**

In this article, we elucidate the causes, symptoms, treatments, and outcomes associated with penile lymphangiomas found in the literature while also presenting the case of a 30-year-old African-American man diagnosed with acquired penile lymphangioma.

## Résumé

**Contexte:**

Les lymphangiomes du pénis sont des manifestations rares des lymphangiomes ou des malformations lymphatiques qui sont plus fréquemment observés dans les régions de la tête ou du cou au niveau du corps. Les lymphangiomes sont aussi classés en lymphangiome circumscriptum, lymphangiome caverneux, hygroma kystique, ou en lymphangiomes acquis (aussi connus sous l’appellation lymphangiectasie), selon leur profondeur et leur étiologie.

**Résultats:**

Une revue de la littérature ne révèle que 30 cas de lymphangiomes du pénis rapportés entre 1947 et Mars 2018. Plusieurs causes ont été attribuées aux lymphangiomes acquis du pénis, parmi lesquelles les traumatismes, le phimosis et les infections. Bien que les lymphangiomes péniens puissent être initialement pris pour une infection, une recherche des antécédents et un examen clinique minutieux sont suffisants pour diagnostiquer cliniquement un lymphangiome du pénis. L’excision chirurgicale a toujours constitué le gold standard du traitement de cette maladie. Lorsqu’elle est asymptomatique, les patients peuvent choisir un traitement conservateur en évitant les seuls traumatismes mécaniques. D’autres médecins ont proposé de nouveaux traitements ayant pour objectif de débarrasser les patients de leurs lymphangiomes péniens, comme la procédure au laser par étapes.

**Conclusion:**

A partir des données de la littérature, nous avons clarifié dans cet article les causes, symptômes, traitements et résultats associés aux lymphangiomes du pénis ; nous avons aussi présenté le cas d’un homme afro-américain âgé de 30 ans diagnostiqué comme porteur d’un lymphangiome pénien acquis.

**Mots-clés:**

Caverneux, Circumscriptum, Kystique, Hygroma, Lymphangiome, Lymphangiectasie, Pénien, Pénis.

## Background

Redenbacher et al. first described lymphangiomas, or “lymphatic malformations,” in 1828 [1]. Since then, lymphangiomas have been classified further in the literature as lymphangioma circumscriptum, cavernous lymphangiomas, cystic hygromas, and acquired lymphangiomas (also known as lymphangiectasias). Lymphangiomas are most commonly found in the head and neck, and two-thirds of all lymphangiomas are found by two years of age. The literature provides detailed pathological, radiographical, and clinical findings associated with lymphangiomas of the head and neck. On the contrary, fewer than 50 cases of lymphangiomas of the penis have been reported since the first description by Ferris et al. in 1944. Presumably, lymphangiomas of the penis are under-reported because the penile lesions, unlike the pathological counterparts in the head/neck, often go unnoticed by not only the physician but also the patient [2]. Furthermore, lymphangiomas of the penis are often misdiagnosed and mistreated as genital warts, molluscum contagiosum or gonorrhea [3]. The objective of this study is to define the different types of penile lymphangiomas by etiology, clinical findings, and treatments. Also, we report a case of acquired lymphangioma of the penis in a 30-year-old male treated successfully by surgical excision.

## Methods

We conducted a literature review identifying all publications using the keywords: “penile” OR “penis” AND “lymphangioma” OR “lymphangiectasis” OR “lymphangiectasia.” Following our institutional protocol, the literature review identified relevant studies via a computer-aided search of American (MEDLINE from 1946 – March 31, 2018) and European articles (Embase 1947 – March 31, 2018). Publications found in languages other than English were excluded. Publications reporting lymphangiomas of the scrotum, perineum, and/or surrounding area not including the penis were excluded. The publications reporting benign transient lymphangiectasis of the penis (BTLP) were also excluded as BTLP, commonly known as sclerosing lymphangitis, is a transient condition that often results after sexual activity and holds minimal medical or surgical relevance. Classifications of penile lymphangioma found in Table 1 corresponding to the case classification from their original publication include “Acquired” (lymphangioma), “Cavernous” (lymphangioma), (lymphangioma) “Circumscriptum,” and (lymphangioma) “Circumscriptum Cysticum” (for cystic hygroma). The classification “Acquired [inferred]” was reserved for cases of lymphangiectasis/lymphangiectasia as well as reported cases of lymphangioma circumscriptum resulting from non-congenital causes. Although controversy exists in the difference between lymphangioma and lymphangiectasis/lymphangiectasia, all cases of lymphangiectasis/lymphangiectasia are reported as “Acquired [inferred]” in Table 1 for simplicity.

**Table 1:** A summary of penile lymphangioma cases reported in the literature

Author	Diagnosis	Age of Onset	Causes/Comorbidities	Location	Treatment	Outcome
Present case	Acquired	30 years old with 21-year history	Skin of penis caught in zipper when patient was 9 years old	Asymptomatic bumps circumferentially around distal shaft of penis	Surgical resection	No pain, erythema or discharge found at 1 month follow-up
Gupta S et al. [11]	Acquired	20 years old	Recurrent swelling with multiple, minute, papulo-vesicular lesions in right foot and leg from age of 3–4 months	Asymptomatic papular lesions on penis and scrotum present for 2–3 months	Simple electrofulguration of visible papulo-vesicles on penis	–
	Acquired	35 years old with 20-year history	–	Papular lesions and gradual swelling of scrotum and penis (shaft, frenulum and around external urethral meatus)	No intervention	–
S Adikari et al. [10]	Acquired	47 years old with 25-year history	Misdiagnosed as genital warts, treated for gonorrhea 5 years after	Smooth to palpation, wart-like lesion on dorsal aspect of penis, otherwise asymptomatic	Surgical excision	Successful with no sign of reccurrence
D. M. Piernick 2nd et al. [8]	Acquired	48 years old with 5-year history	Hidradenitis suppurativa of buttocks, gluteal cleft and perineal area	Asymptomatic, multiple semitranslucent skin colored papules coalescing into plaques on penile shaft, scrotum and perineum	None	–
Errichetti et al. [4]	Acquired	61 years old with 1-year history	Severe phimosis	Constricting phimotic ring and considerable edema of glans and distal foreskin with several translucent preputial papulovesicles (some slightly hyperkeratotic) localized close to balanopreputial sulcus	None - patient was waiting for surgery to correct phimosis – no follow-up information was provided	–
Zhang et al. [6]	Acquired	8 years old with 2-week history	Surgery to correct phimosis	Asymptomatic, multiple small vesicular lesions on glans	“watch and wait” policy	Lesions resolved in 3 weeks
Dehner LP. et al. [21]	Acquired	39 years old with 6-week history	–	Shaft, dorsum of penis	Surgical excision	Successful with no sign of recurrence
Ferris et al. [2]	Acquired	35 years old with 30-year history	Measles? Circumcision? Pneumonia? All illnesses exacerbated condition	Lesions on foreskin of penis, scrotum and adjacent areas of the thigh and perineum	Surgical excision with skin graft	Successful with no sign of recurrence
Hagiwara et al. [9]	Acquired	65 years old with 18-year history	Filariasis	Scrotum, extending to foreskin	Surgical excision and skin grafting	Successful with no sign of recurrence - transient penile edema present for few weeks
Sadikoglu et al. [5]	Acquired (inferred)	15 years old with 3-year history	Blunt trauma caused skin thickening	Penile and scrotal skin	Surgical excision and skin grafting	Successful with no sign of recurrence
Kokcam et al. [19]	Acquired (inferred)	19 years old with 3-year history	–	Multiple translucent and hemorrhagic vesicles on shaft and glans of penis. Surface was smooth, some umbilicated	Pt refused surgical intervention, advised to avoid mechanical trauma, apply silver sulfadiazine cream to ruptured lesions	No new lesions, overall number of lesions declined markedly with no other complications
Latifoglu et al. [22]	Acquired (inferred)	10 years old with 6-year history	–	Penoscrotal lymphedema with erythematous plaque (irregular, well-defined border) on penile shaft and gelatinous-appearing, coalescent, verrucous vesicles and papules on scrotum	Surgical resection	Successful with no sign of recurrence
Maloudijan et al. [26]	Acquired (inferred)	50 years old with 10-year history	–	Asymptomatic, 2 mm large vesicular lesions in sulcus coronarius from adjacent foreskin and glans	Patient abstained	–
Cestaro et al. [23]	Acquired (inferred)	24 years old	HPV - genotype 6 comorbidity	Lesions on inguinal area, scrotum, penis, glans with associated edema of penis and lips	Surgery	Successful with no recurrence
Shi G. et al. [7]	Acquired [inferred]	23 years old with 40-day history	Circumcision following phimosis 5 years ago	Asymptomatic translucent, yellowish, elevated, thick walled cystic lesions on right side of glans	2940 nm nonablative fractional Er:YAG laser at 2–3 week intervals with power density of 3 J/cm^2^ at 20 ms and a 5 mm spot size	Lesions disappeard obviously after 4 sessions, no recurrence, dyspigmentation and paresthesia
Shah A. et al. [24]	Acquired [inferred]	11 years old with few month history	–	Asymptomatic, soft mass on dorsal aspect of penis with extension towards right hemiscrotum	Local surgical resection	Recurrence 11 months following surgery
Bardazzi et al. [25]	Acquired [inferred]	45 years old	–	Sulcus of prepuce	Diathermy	Successful with no sign of recurrence
Llanes et al. [16]	Cavernous	20 years old	–	Soft lesion in dorsal area of prepuce	Circumcision	Successful with no sign of recurrence
Hayashi et al. [17]	Cavernous	32 years old	–	Tumor on coronary sulcus of glans and submucosa	–	–
	Cavernous	35 years old	–	Tumor on coronary sulcus of glans and submucosa	–	–
Geuekdjian et al. [27]	Circumscriptum	3 years old	Congenital [inferred]	Asymptomatic, edematous swelling of penis particularly in skin spreading upwards to left groin	En bloc resection	Successful with no sign of recurrence
Demir et al. [18]	Circumscriptum	21 years old with history since childhood	Congenital [inferred]	Recurrent infections, drainage of vascular lesions, penoscrotal deformity and inability to have sexual intercourse	Surgical excision	Successful, no sign of remission
Ferro et al. [3]	Circumscriptum	16 years old	Congenital [inferred]	Tense vesicles filled with clear fluid on coronal region	3 surgeries - remission every time. Denuded penis buried in tunnel guided through scrotum, 6 months after - shaft lift and recreated with scrotal skin	No negative consequences, local hairiness treated cosmetically
Osborne et al. [14]	Circumscriptum	45 years old	Lichen planus - treated with cryotherapy	Cluster of translucent vesicles on shaft of penis and coronal sulcus. Balanomegaly.	Treatment declined	–
Tsur et al. [28]	Circumscriptum	8 month old	Congenital [inferred]	asymptomatic elevated lesions on glans penis around meatus and dorsal aspect of penis	Surgical excision	Successful with no sign of recurrence
Drago et al. [12]	Circumscriptum	27 years old	Ulceritive colitis		–	–
Handa et al. [29]	Circumscriptum	10 years old with 9 year history	Congenital [inferred]	Penis, scrotum, groins bilaterally	–	–
Swanson et al. [13]	Circumscriptum	16 years old	Recurrent cellulitis of the penis and scrotum	Subcutaneous tissue of penis proximal to glans and skin of left proximal scrotum	–	–
Greiner et al. [15]	Circumscriptum cysticum	13 years old	Congenital malformation	Edematous thickening of penile and scrotal skin	–	–

A summary of penile lymphangioma cases reported in the literature

## Case presentation

**Fig. 1:**
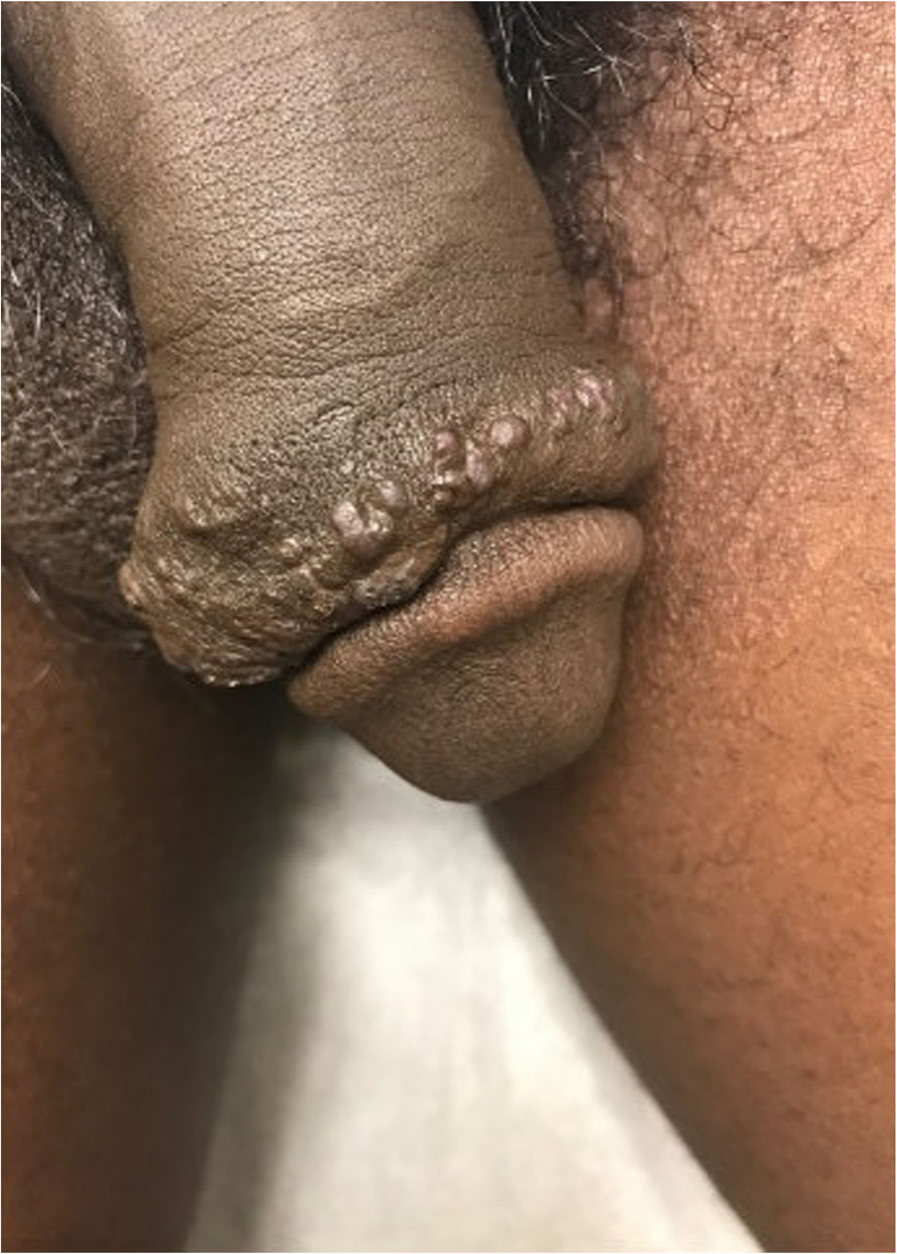
Clinical examination of patient’s penile shaft and glans: Multiple firm skin-colored papules, some with exophytic crusting and underlying edema, present on the right side of the patient’s penile shaft, immediately proximal to the glans

**Fig. 2:**
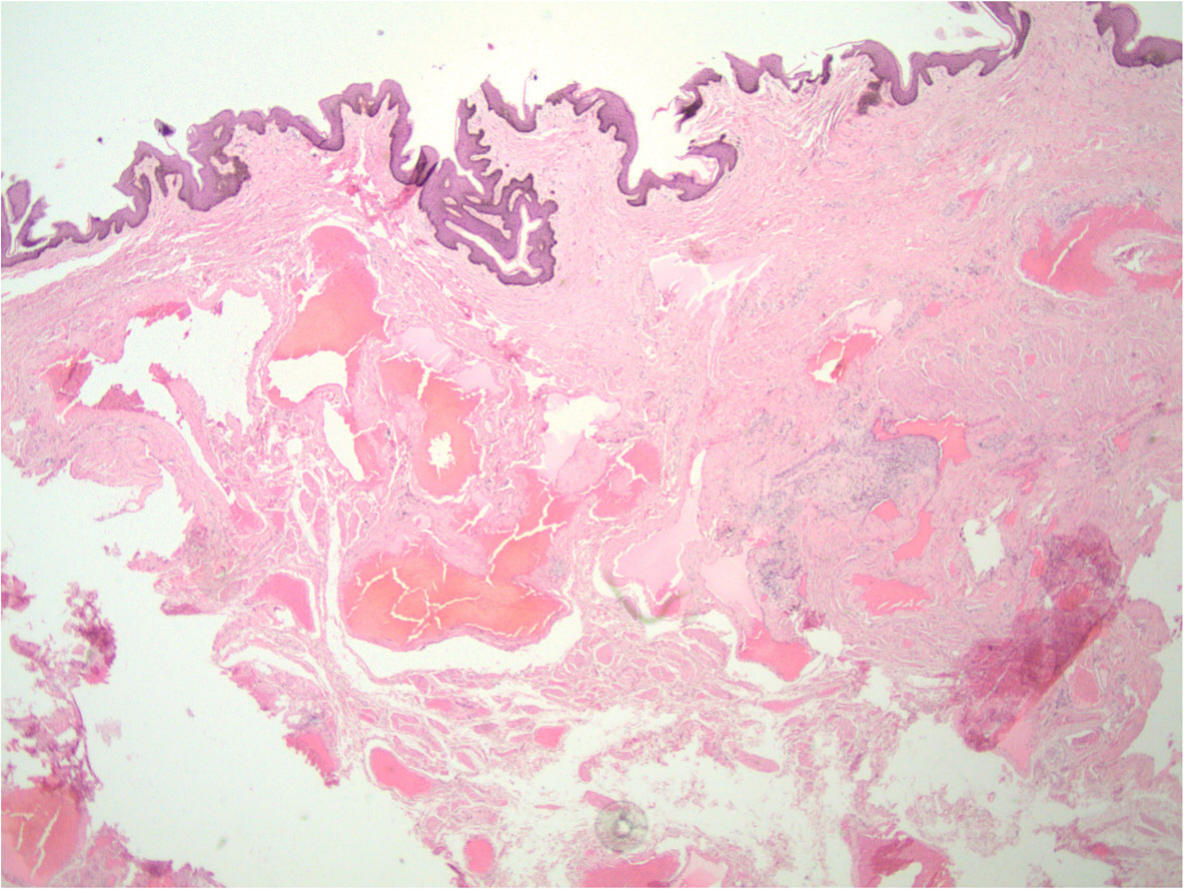
Histopathological image following biopsy of patient’s penile lesions: Histopathological staining from biopsy by punch technique of distal dorsal penile shaft shows dilated vascular channels consistent with benign lymphangioma of the penis

A 30-year-old African-American male presented to his primary care physician with a chief complaint of a several-year history of unhealing wounds on the right side of his penile shaft after his penis was caught in his zipper several years ago. Patient became concerned after noting white penile discharge 2 weeks prior. He denies any anal or oral lesions as well as exposure to any sexually transmitted diseases (STDs). Following outpatient specialty referral, the dermatologist reported excess skin tissue with underlying edema circumferentially on the distal penile shaft with overlying multiple firm skin-colored papules, some with exophytic crusting [Fig. 1]. Chlamydia trachomatis, human immunodeficiency virus (HIV), Neisseria gonorrhea and syphilis were negative. Subsequent biopsy found dilated vascular channels consistent with benign acquired lymphangioma of the penis **(**Fig. 2**)**, and the patient was referred to urology for evaluation and management. With the urologist, the patient elected for surgical intervention due to cosmetic concerns despite the asymptomatic nature of the lymphangioma.

The patient underwent circumcision for redundant prepuce, excision of the skin lesion and penile foreskin reconstruction. A circumferential incision was made on the mucosa 0.5 cm proximal to the glans, distal to the lymphangioma. The foreskin was then retracted and another circumferential incision was made around the mucosal skin. The foreskin was then dissected using Bovie cautery and blunt dissection while the foreskin with lymphangioma tissue was excised. Intraoperative and postoperative courses were unremarkable. At 1-month follow-up, the patient reported no pain, erythema or discharge from the wound.

## Results

The current literature review identified 27 cases in 25 publications. In the largest group, the 18 acquired lymphangiomas of the penis identified in the literature review encompassed a wide variety of cases. One was attributed to phimosis and 2 to trauma [4, 5]. Two iatrogenic cases resulted from correction of phimosis [6, 7]. One infectious case was attributed to filariasis (1 case), and 1 inflammatory case was attributed to hidradenitis suppurativa (1 case) [8, 9]. Two idiopathic cases were previously mistaken for genital warts and gonorrhea [10, 11].

Lymphangioma circumscriptum of the penis in this literature review was reported in eight, five of which were ruled to be congenital. The other three were notable for comorbidities of ulcerative colitis, recurrent cellulitis, and Lichen planus [12–14]. The only case of lymphangioma circumscriptum cysticum or cystic hygroma was found to have a congenital origin [15]. Lastly, cases of cavernous lymphangioma were attributed to a tumor (2 cases) and one with an unknown cause [16, 17].

## Discussion

Lymphangiomas are generally described as uncommon, hamartomatous malformations of the lymphatic system. Based on depth and size of the lymph vessels, classifications include lymphangioma circumscriptum (most superficial), cavernous lymphangioma and cystic hygroma (most deep). Further subdivisions derive from perceived cause. Congenital lymphangiomas are thought to result from fetal lymph vessels that failed to involute and/or failed to join with the central lymphatic system. On the contrary, acquired lymphangiomas may result from trauma, certain infections (cellulitis, neoplastic disease, tuberculosis, filariasis), radiotherapy, pregnancy, scleroderma, severe phimosis or STDs [10].

Although the causes of these various types of lymphangioma may differ, the clinical presentation is often similar. The majority of the penile lymphangiomas present as asymptomatic, fluid-filled, translucent lesions or vesicles most commonly on the shaft or coronal sulcus of the penis. Symptomatic lesions, on the other hand, typically focus on sexual dysfunction. One case of acquired lymphangioma presented with severe phimosis and inability to produce an erection [4]. Also, one case of lymphangioma circumscriptum caused recurrent infection, intermittent drainage and sexual inactivity [18]. Given the similarities in clinical presentation, a proper diagnosis becomes contingent on a thorough history and physical examination in the case of lymphangioma of the penis. To that end, clinicians seeking to properly diagnose and treat lymphangiomas of the penis must effectively rule out certain infectious diseases such as molluscum contagiosum and gonorrhea [19]. This is generally possible following a thorough history and physical examination alone. However, in some rare cases, a biopsy with accompanying pathology report or an infectious disease workup may be necessary. Dermatology consultation may be selected for unclear cases. Interestingly, Errichetti et al. recently elucidated the potential role of dermoscopy in the diagnosis of penile lymphangioma by describing the presence of “yellowish-reddish, well-demarcated, round or oval lacunae surrounded by whitish areas or lines,” which may be common characteristics of lymphangiomas [4, 20]. This observation warrants further investigation into the utility of dermoscopy as a quick, non-invasive method of definitively diagnosing lymphangiomas.

Many authors may argue that lymphangiomas of the penis do not always require treatment, given the mostly asymptomatic nature of these lesions. However, patients may request intervention for cosmetic reasons. Of the 18 acquired lymphangiomas reported in Table 1 (including the present case), 9 were treated by surgical excision [2, 5, 9, 10, 21–23] – only one of which recurred 11 months following surgery [24]. And, one case reported an electrofulguration (or electrocautery) of the visible papulo-vesicles on the penis [11]. Shi et al. found that the acquired lymphangioma of the penis was amenable to 2940-nm Erbium-doped Yttrium Aluminum Garnet laser once every 2–3 weeks, wherein the lesions disappeared after the fourth session with no evidence of recurrence, dyspigmentation or paresthesia [7]. Bardazzi et al. utilized high-frequency electric currents, called diathermy, to successfully obliterate the acquired lymphangioma without evidence of recurrence [25]. On the other hand, Zhang et al. advocated for a “watch and wait” policy that interestingly allowed the lesions to self-heal within three weeks time [6]. Lymphangiomas that develop following circumcision may spontaneously resolve. Abstinence from treatment was observed in two patients, one of whom opted for protecting the lesions from mechanical trauma and applying a silver sulfadiazine cream to any ruptured lesions [19, 26]. This management decreased the number of existing lesions and prevented new lesions. Finally, one patient was waiting for surgery to correct phimosis; no follow-up information was provided [4]. Neither treatment nor follow-up was documented in the remaining two cases [8, 11].

Of the 3 cavernous lymphangiomas reported in Table 1, one underwent circumcision with no sign of recurrence following the treatment [16]. The other two cases failed to specify treatment [17]. Of the 8 cases of lymphangioma circumscriptum of the penis, 3 underwent surgical resection successfully with no signs of recurrence [18, 27, 28]. Another patient with lymphangioma circumscriptum (Table 1) underwent 3 surgeries with recurrence every time. For the fourth operation the physicians adopted a more radical surgical approach, in which the penile shaft was denuded and buried in the scrotum [3]. Six months later, the penile shaft was raised, and the skin was reconstructed using a scrotal graft. The outcome of this procedure was favorable with the only noteworthy complication of growth of transposed hair, treated cosmetically. Of the remaining 4 lymphangioma circumscriptum cases shown in Table 1, treatment was denied for 1, and the remaining 3 had no information on treatment or outcome [12–14, 29].

## Conclusions

In summary, penile lymphangioma can be divided into four categories: 1) acquired, 2) lymphangioma circumscriptum, 3) cavernous lymphangioma, and 4) cystic hygroma. Commonly mistaken for infectious lesions, lymphangiomas underscore the importance of an appropriate history and physical to properly identify and treat the lymphatic malformation.

## Abbreviations

BTLP: Benign transient lymphangiectasis of the penis
HIV: Human immunodeficiency virus
STDs: Sexually transmitted diseases
